# Effectiveness of Ritonavir-Boosted Protease Inhibitor Monotherapy in Clinical Practice Even with Previous Virological Failures to Protease Inhibitor-Based Regimens

**DOI:** 10.1371/journal.pone.0148924

**Published:** 2016-02-12

**Authors:** Luis F. López-Cortés, Manuel A. Castaño, Miguel A. López-Ruz, María J. Rios-Villegas, José Hernández-Quero, Dolores Merino, Patricia Jiménez-Aguilar, Manuel Marquez-Solero, Alberto Terrón-Pernía, Francisco Tellez-Pérez, Pompeyo Viciana, Francisco Orihuela-Cañadas, Zaira Palacios-Baena, David Vinuesa-Garcia, Jose M. Fajardo-Pico, Alberto Romero-Palacios, Guillermo Ojeda-Burgos, Juan Pasquau-Liaño

**Affiliations:** 1 Instituto de Biomedicina de Sevilla, Hospital Universitario Virgen del Rocío/CSIC/Universidad de Sevilla, Sevilla, Spain; 2 Hospital Universitario Carlos Haya, Málaga, Spain; 3 Hospital Universitario Virgen de las Nieves, Granada, Spain; 4 Hospital Universitario Virgen Macarena, Sevilla, Spain; 5 Hospital Universitario San Cecilio, Granada, Spain; 6 Complejo Hospitalario Juan Ramón Jiménez, Huelva, Spain; 7 Hospital Universitario de Puerto Real, Cádiz, Spain; 8 Hospital Universitario Virgen de la Victoria, Málaga, Spain; 9 Hospital Jerez de la Frontera, Cádiz, Spain; 10 Hospital La Línea, Cádiz, Spain; University of Pittsburgh Center for Vaccine Research, UNITED STATES

## Abstract

**Background and Objective:**

Significant controversy still exists about ritonavir-boosted protease inhibitor monotherapy (mtPI/rtv) as a simplification strategy that is used up to now to treat patients that have not experienced previous virological failure (VF) while on protease inhibitor (PI) -based regimens. We have evaluated the effectiveness of two mtPI/rtv regimens in an actual clinical practice setting, including patients that had experienced previous VF with PI-based regimens.

**Methods:**

This retrospective study analyzed 1060 HIV-infected patients with undetectable viremia that were switched to lopinavir/ritonavir or darunavir/ritonavir monotherapy. In cases in which the patient had previously experienced VF while on a PI-based regimen, the lack of major HIV protease resistance mutations to lopinavir or darunavir, respectively, was mandatory. The primary endpoint of this study was the percentage of participants with virological suppression after 96 weeks according to intention-to-treat analysis (non-complete/missing = failure).

**Results:**

A total of 1060 patients were analyzed, including 205 with previous VF while on PI-based regimens, 90 of whom were on complex therapies due to extensive resistance. The rates of treatment effectiveness (intention-to-treat analysis) and virological efficacy (on-treatment analysis) at week 96 were 79.3% (CI_95_, 76.8−81.8) and 91.5% (CI_95_, 89.6–93.4), respectively. No relationships were found between VF and earlier VF while on PI-based regimens, the presence of major or minor protease resistance mutations, the previous time on viral suppression, CD4^+^ T-cell nadir, and HCV-coinfection. Genotypic resistance tests were available in 49 out of the 74 patients with VFs and only four patients presented new major protease resistance mutations.

**Conclusion:**

Switching to mtPI/rtv achieves sustained virological control in most patients, even in those with previous VF on PI-based regimens as long as no major resistance mutations are present for the administered drug.

## Introduction

The idea of simplifying the HIV-1 antiretroviral treatment (ART) once achieved virological suppression arose after the initial enthusiasm that the efficacy of the first highly active antiretroviral therapies was tempered by their short- and long-term toxicity and the frequent occurrence of resistance-associated mutations. Nevertheless, this strategy failed to sustain viral suppression when compared with maintaining triple-drug therapy in the earlier studies, probably due to the low genetic barrier and/or the low antiviral potency of the drugs used at that time [1–3].

Years later, the idea re-emerged after researchers became aware of the potent antiviral activity and the high genetic barrier of the ritonavir-boosted protease inhibitors [4]. Since then, a considerable amount of data have been accumulated on ritonavir-boosted protease inhibitor monotherapy (mtPI/rtv), particularly for lopinavir/ritonavir (mtLPV/rtv) and darunavir/ritonavir (mtDRV/rtv), from several clinical trials in which a high proportion of the patients maintained undetectable viremia with these simplified regimens [4–12]. However, significant controversy still exists regarding mtPI/rtv as a maintenance strategy [13–17]. Moreover, until now, at most, mtPI/rtv has only been administered to patients without a history of virological failure (VF) while on prior protease inhibitor-based therapy regimens.

Both the encouraging results of the above-mentioned clinical trials and the benefits of simpler regimens lacking the toxicity of nucleoside analogs or other antiretroviral drugs and cost-effectiveness [18,19] have made the use of mtPI/rtv a frequent practice in Spain, particularly in Andalusia. In fact, this treatment strategy is considered as a simplification option in both the Spanish and European guidelines for the use of antiretroviral agents in HIV-1-infected adults from 2009 onwards, although applying only to patients without history of failure on prior PI-based therapy and who have had viral load < 50 copies/mL in at least the past 6 months [14,15,20,21]

In this study, we evaluated the treatment effectiveness of two mtPI/rtv regimens in an actual clinical practice in the largest cohort reported to date, including patients that experienced previous virological failures while on protease inhibitors (PIs).

## Patients, Material and Methods

### Study population and design

In this retrospective study, all of the HIV-infected adults at the participating centers who were switched from a triple antiretroviral regimen to either mtLPV/rtv (400/100 mg twice daily) or mtDRV/rtv (800/100 mg once daily) for the first time, from January 2010 to September 2012, were consecutively included. The participating centers (sorted by number of cases included) were Hospital Universitario Virgen del Rocío (Sevilla), Hospital Universitario Carlos Haya (Málaga), Hospital Universitario Virgen de las Nieves (Granada), Hospital Universitario Virgen Macarena (Sevilla), Hospital Universitario San Cecilio (Granada), Complejo Hospitalario Juan Ramón Jiménez (Huelva), Hospital Universitario de Puerto Real (Cádiz), Hospital Universitario Virgen de la Victoria (Málaga), Hospital Jerez de la Frontera (Cádiz), Hospital La Línea(Cádiz). The authors were the attendant physicians involved in making treatment decisions for patients. In most of these centers, clinical data of HIV-infected patients are recorded prospectively using the same software application (ACyH®, Betek 43 SL, Spain). The criteria for switching from a triple antiretroviral regimen to mtPI/rtv were fairly uniform across the participating centers. As a rule, patients were switched to mtPI/rtv after ≥6 months of plasma HIV-RNA determinations of <50 copies/mL and a treatment history of no previous virological failure (VF) while on PI-based regimens. In cases in which the patient had an earlier VF while on PI-based regimens, lack of major HIV resistance mutations to LPV/rtv (V32I, I47V/A, L76V, and V82A/F/T/S) or DRV/rtv (I47V, I50V, I54M/L, L76V, and I84V), respectively, in the genotypic resistance tests was mandatory according to the 2010 International AIDS Society [22]. Patients were switched from a triple regimen to boosted monotherapy because of adverse events (AEs) from the previous regimen or to simplify the treatment approach. However, not all patients who met the inclusion criteria at the participating centers were switched from triple therapy to mtPI/rtv. It largely depended on the decision of the responsible physicians, which was influenced by their confidence in mtPI/rtv and the acceptance by the patient after receiving verbal information. MtLPV/rtv was used more frequently in the first part of the study, while mtDRV/rtv was used more recently as data of clinical trials on that drug were reported.

No entry restrictions were made regarding plasma HIV-RNA, CD4 T cell count, illegal drug or methadone use, HCV-coinfection, or alterations in laboratory parameters. MtPI/rtv was not prescribed in cases of pregnancy, hepatitis B coinfection, or concomitant use of drugs with potential interactions with lopinavir/ritonavir or darunavir/ritonavir, respectively. The assembling of data was approved by the Ethics Committee in Biomedical Research of Andalucía and the Spanish Agency for Medicines and it was conducted according to the rules of the Declaration of Helsinki. The ethics committee specifically waived the need for patient consent since, according to Spanish law, the study did not require informed consent due to its retrospective nature and the fact that only completely anonymous information from existing records was collected, thereby ensuring the protection of personal data in accordance with the Personal Data Protection Organic Law15/199 enacted on December 13, 1999.

### Endpoints, follow-up, and assessments

The primary clinical endpoint was treatment effectiveness, which was measured as the percentage of patients with virological suppression after 96 weeks according to intention-to-treat analysis (non-complete/missing = failure). VF was defined as a confirmed plasma HIV-RNA of >200 copies/mL, considering the time of the first assessment meeting the failure criteria as the time of failure, or a single HIV-RNA level >200 copies/mL, if followed by loss of follow-up. A cut-off level of 200 copies/mL was chosen because it is a more accurate measurement of VF than a lower cut-off value [12,23,24]. As a secondary outcome, virological efficacy was assessed using on-treatment (OT) analysis. Additionally, patients were classified into four non-overlapping groups based on their virological outcome during the follow-up: i) continuous viral load of <50 copies/mL (cUV); ii) blips: transitory episodes of HIV-RNA viral loads of >50 copies/mL, preceded and followed by a plasma viral load of <50 copies/mL without changes in the antiretroviral treatment; iii) intermittent viremia (IV): episodes of detectable plasma HIV-RNA viral loads of >50 copies/mL during the follow-up without meeting blip or VF criteria; and iv) VF as defined above. AEs were categorized via the standardized toxicity-grade scale used by the AIDS Clinical Trials Group [25]. However, in patients with chronic viral hepatitis C or cirrhosis, toxicity was classified according to changes relative to the baseline values rather than the ULN: grade 0, <1.25 x baseline; grade 1, (1.25 to 2.5) x baseline; grade 2, (2.6 to 3.5) x baseline; grade 3, (3.6 to 5) x baseline; and grade 4, >5 x baseline.

Patient assessment was performed at baseline and every three months thereafter, and included AEs, biochemical profiles, hematologic counts, flow cytometric counts of CD4^+^ T cells, and plasma HIV-RNA levels measured by polymerase chain reaction (PCR) (Amplicor HIV-1 Monitor test v. 1.0 with a lower detection limit of 50 copies/ml or COBAS AmpliPrep/COBAS TaqMan HIV-1 Test, v. 2.0 with a lower detection limit of 15 copies/ml, more recently). Patients missing two consecutive scheduled visits were considered lost to follow-up.

### Statistical analysis

Descriptive statistics were used for demographic, epidemiological, and clinical data, prior ART, CD4 T cell count, and plasma HIV-RNA levels. Quantitative and qualitative variables are expressed as median, interquartile range (IQR), or range and number (%), respectively. Categorical variables were compared by using Student’s t-test or the Mann-Whitney non-parametric test, according to their distribution. The relationships between VF and the different variables were assessed by the χ^2^ test or Fisher's exact test and Spearman's rank correlation coefficients. Time-to-event analyses were performed using Kaplan-Meier survival curves and the log-rank test.

## Results

### Baseline patient characteristics

A total of 1096 patients were initially included in the study, 36 of them were excluded from the analysis due to an HIV-RNA of >50 copies/ml at the time of the mtPI/rtv switching (n = 32) and four because the presence of one major protease resistance mutation for lopinavir/ritonavir (V82A/T) in previous genotypic resistance tests. Out of the remaining 1060 patients, 88.2% were switched to mtPI/rtv for simplification and 11.8% were switched due to AEs from the previous regimen. The main baseline characteristics of the patients are shown in Table 1, categorized according to the type of PI used (LPV/rtv or DRV/rtv). Before switching to mtLPV/rtv, almost 90% of the patients were on ART with two nucleos(t)ide reverse transcriptase inhibitors (NRTIs) plus one ritonavir-boosted protease inhibitor, mostly LPV/rtv (76.0%). In contrast, the previous ART regimens were more heterogeneous in the mtDRV/rtv group, in which only 101 patients (25.4%) were on a DRV/rtv-based treatment before switching to mtDRV/rtv. It is important to note that 90 patients were on complex combinations, including one NRTI or one non-nucleoside reverse transcriptase inhibitor (NNRTI) plus raltegravir plus one PI/rtv or even enfuvirtide plus PI/rtv ± maraviroc, due to extensive resistance to NRTIs and/or NNRTIs (Table 1).

**Table 1:** Baseline patients’ characteristics.

	mtDRV/rtv	mtLPV/rtv	
	n = 596	n = 464	*p*
Male, no. (%)	446 (74.8)	344 (74.1)	0.832
Age, years. M (range)	46 (18–88)	46 (18–77)	0.189
Weight, kg. M (range)	70 (39–115)	68 (40–123)	0.210
Risk factor for HIV, no. (%)			0.024
Heterosexual	161 (27.0)	118 (25.4)	
Homosexual	171 (28.7)	96 (20.7)	
Other	24 (4.0)	16 (3.4)	
Drug addiction or methadone treatment	61 (10.2)	57 (12.3)	0.617
Previous C stage (CDC), no. (%)	164 (27.5)	158 (34.1)	0.027
Nadir CD4^+^ T cells count/μL. M (range)	174 (1–861)	155 (1–880)	0.517
CD4^+^ T cells count/μL. M (range)	585 (46–1346)	585 (76–1638)	0.431
Chronic hepatitis, no. (%)	198 (33.2)	214 (46.1)	0.001
Cirrhosis	27 (5.3)	33 (7.1)	0.106
No. of previous antiretroviral regimens	4 (1–10)	4 (1–10)	0.170
Months on antiviral therapy. M (range)	96 (9–288)	78 (9–238)	0.051
Previous failure on PI, no. (%)	123 (20.6)	82 (17.6)	0.393
Previous failure on NNRTIs, no. (%)	123 (21.2)	90 (19.4)	0.825
Months with HIV-RNA <50 copies/mL, M (range)	34 (6–173)	30 (6–184)	0.451
Blips in the previous 12 months, no. (%)	141 (23.6)	70 (15.0)	0.001
Last ART combinations, no. (%)			
2 NRTIs + PI/rtv	397 (66.6)	410 (8.3)	0.001
LPV/rtv	81 (20.4)	312 (76.0)	
SQV/rtv	91 (22.9)	78 (19.0)	
DRV/rtv	101 (25.4)	2 (0.5)	
fAPV/rtv	65 (16.3)	9 (2.2)	
ATV/rtv	59 (14.8)	9 (2.1)	
2 NRTIs + NNRTIs	136 (22.8)	21 (4.5)	0.001
2 NRTIs + RAL or MRV	6 (1.0)	‒	
Last 2 NRTIs used			
TDF + FTC	298 (55.3)	232 (53.8)	0.540
ABV + 3TC	134 (24.9)	78 (18.1)	
Other 2 NRTIs	107 (19.9)	121 (28.1)	
Other combinations	57 (9.5)	33 (7.1)	0.155
NRTI + NNRTI + PI/rtv	9	5	
NRTI + RAL + PI/rtv	17	18	
NNRTI + RAL + PI/rtv	7	2	
NNRTI + MRV + PI/rtv	2	‒	
NNRTI + PI/rtv	6	2	
RAL + PI/rtv	13	5	
MRV + PI/rtv	2	‒	
T20 + PI/rtv	1	‒	
T20 + RAL + PI/rtv	‒	1	

mtDRV/rtv, darunavir/ritonavir monotherapy. mtLPV/rtv, lopinavir/ritonavir monotherapy. M, median; ART, antiretroviral therapy; NRTIs, nucleos(t)ide reverse transcriptase inhibitors; PI, protease inhibitors; PI/rtv, ritonavir-boosted protease inhibitors; NNRTIs, non-nucleoside reverse transcriptase inhibitors; LPV/rtv, lopinavir/ritonavir; SQV/rtv, saquinavir/rtv; DRV/rtv, darunavir/ritonavir; fAPV/rtv, fosamprenavir/ritonavir; ATV/rtv, atazanavir/ritonavir; NNRTIs, non-nucleoside reverse transcriptase inhibitors; RAL, raltegravir; MRV, maraviroc; T20, enfuvirtide.

Two hundred and five patients (19.3%) had a previous VF on either a non-ritonavir-boosted protease inhibitor-based regimen (41.9%), a PI/rtv-based regimen, or both (58.1%). One hundred and fifty eight (77.1%) of these 205 patients had available genotypic resistance tests just after these VFs; in 107 of those patients, the HIV protease gene showed a wild-type virus or only minor protease mutations while in 54 of them one or two major protease mutations plus minor mutations were observed. Although no patients had major resistance mutations for lopinavir or darunavir, 37 of the patients receiving mtLPV/rtv had minor mutations for that drug (median, 2; range, 1–3) and eight patients receiving mtDRV/rtv had minor mutations for darunavir/ritonavir (median, 2; range, 1–4) (Table 2).

**Table 2:** Mutations in HIV protease gene after previous virological failure (VF) on protease inhibitor-based regimen.

Protease mutations after previous VF on PI-based regimen (n = 158/205)	n (%)
Wild type	59 (37.3)
Only mm (M, 2; range, 1–7)	48 (30.3)
1 MM (L90M, 25; D30N, 11; V82A, 2; N88S, 2; I50L/V, 2) + mm (M, 3; range, 0–8)	42 (26.6)
2 MM (V82A + L90M, 5; I84V + L90M, 2; N88S + L90M, 2; V82A + I84V, 1; D30N	
+ N88S, 1; I54L + L90M, 1;) + mm (M, 6; range, 4–10)	12 (8.6)

PI, protease inhibitor. mm, protease minor mutations. MM, HIV protease major mutations; M, median. Specific major protease mutations in brackets.

### Treatment effectiveness and safety

Given that the effectiveness rates were similar in the two groups, the data from all of the patients were analyzed together. The Kaplan-Meier estimations of treatment effectiveness by intention to treat analysis were 88.3% (CI_95_, 86.4‒89.4) and 79.3% (CI_95_, 76.8–81.8) at week 48 and 96, respectively.

In addition to VF episodes, other treatment failures were due to: (1) AEs (n = 35; diarrhea, 17; hyperlipidemia, 9; abdominal discomfort or vomiting, 5; increased aminotransferase levels, 2; hyperglycemia, 1; lipohypertrophy, 1); (2) loss to follow-up or treatment dropout (n = 33); (3) death not related to treatment (n = 7); (4) moved to another city or imprisonment (n = 10); and (5) medical decision without VF criteria or AEs (50 patients).

Only four out of the 85 patients with treatment failures due to AEs, loss to follow-up or dropout, deaths, and moving, had detectable viral load at the time of the last available HIV-RNA assessment (59–189 copies/mL). Among the 50 patients changing treatment for medical reasons, 16 had an undetectable viral load at the time of switching. The remaining 34 patients had median HIV-RNA levels of 131 copies/mL (range, 63–491).

The median increase in CD4^+^ T-cell counts from baseline to week 48 and 96 was 32 cells/μL (IQR, -71 to 143) and 49 (IQR, -70 to 170), respectively, as this was inversely proportional to the baseline CD4 counts (r = -0.302 and -0.286; p <0.001). Aminotransferase level increases throughout the follow-up period occurred in 107 (16.5%) out of 648 patients with no previous history of chronic hepatitis [grade 1, 78 patients (12.0%); grade 2, 19 patients (2.9%); grade 3, 8 patients (1.2%), and grade 4, 2 patients (0.3%)], and in 194 (47.0%) out of 412 patients with chronic hepatitis or cirrhosis [(grade 1, 161 patients (39.0%), grade 2, 22 patients (5.3%), grade 3, 7 patients (1.7%), and grade 4, 8 patients (1.9%)]. These alterations were transient and improved without treatment modification in most cases, but it motivated a treatment change in three patients. Regarding the lipid profile, the median (IQR) changes in total cholesterol, HDL-cholesterol, LDL-cholesterol, and triglycerides (mg/DL) for 928 patients at 48 weeks on mtPI/rtv were 11 (-13 to 34), 1 (-5 to 9), 9 (-11 to 29), and 5 (-36 to 55) respectively. After 96 weeks, these values were 12 (-17 to 37), 3 (-3 to 11), 11 (-14 to 30), and 5 (-51 to 48), respectively.

### Virological efficacy and genotypic resistance tests at failure

**Fig 1:**
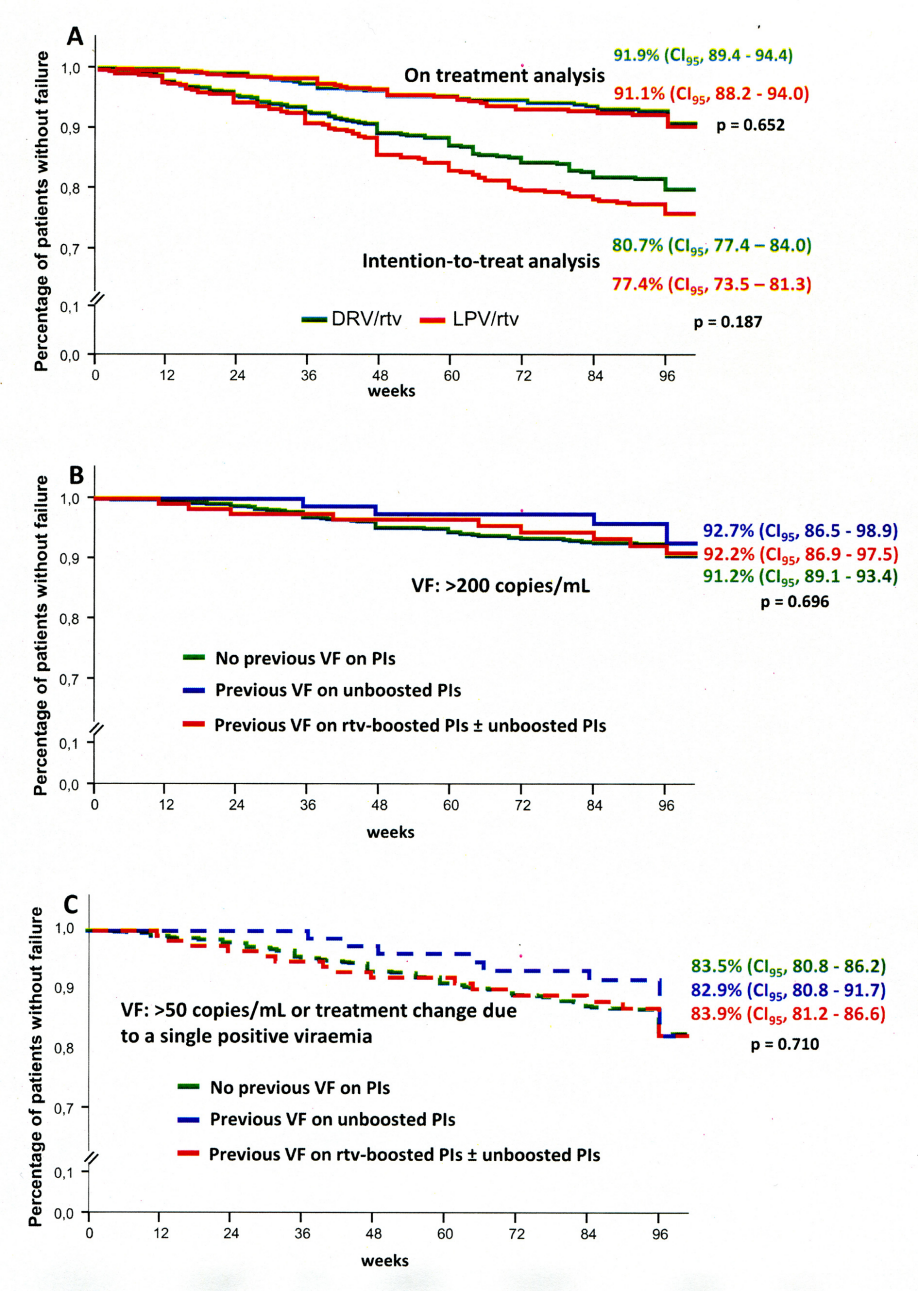
Efficaccy rates. A) Efficacy rates according to the ritonavir-boosted protease inhibitor used by intention-to-treat and by on-treatment analyses. B) Virological efficacy rates according to virological failure (VF) defined as >200 copies/mL or C) >50 copies/mL or treatment change due to a single positive viremia, according to the presence or absence of previous VF on a non-boosted protease inhibitor- and/or ritonavir-boosted protease inhibitor-based regimen (PI/rtv). DRV/rtv, darunavir/ritonavir; LPV/rtv, lopinavir/ritonavir.

The Kaplan-Meier estimations of virological efficacy determined by on-treatment analysis were 95.6% (CI_95_, 94.5–96.7) and 91.5% (CI_95_, 89.6–93.4) at 48 and 96 weeks, respectively (Fig 1A). After 96 weeks there were 39 (6.5%) and 35 (7.5%) VF episodes in the mtDRV/rtv and mtLPV/rtv groups, respectively (p = 0.545). The VF rates were observed to be similar among those patients with and without a previous VF while on a protease inhibitor-based regimen (7.3% vs. 5.9%; p = 0.544) (Fig 1B). No relationships were observed between VF and either the presence of major or minor protease resistance mutations or the total number of both types of mutations. The time period with undetectable viral load before switching to mtPI/rtv was similar between the patients presenting with VF (median, 29 months; range, 6–146) and those who did not present with VP (median, 32 months; range, 6–184), p = 0.961). Likewise, there were no differences between both groups regarding the CD4^+^ T-cell nadir levels (median, 136 cells/μL; IQR, 35–227; range, 1–633 vs. 168 cells/μL; IQR, 64–265; range, 1–880), (p = 0.195). Only one of the 90 patients receiving previous complex therapy due to extensive resistance had a VF. Table 3 and Fig 1C show the estimated VF rates using tighter criteria for VF to compare this study’s results with the findings from other studies with different VF criteria.

**Table 3:** Kaplan-Meier estimations of the efficacy as determined by on-treatment analysis applying different criteria for virological failure.

	Virological efficacy	
Virological failure definition	week 48	week 96
HIV-RNA >200 copies/mL x2 or >200 x1 followed by loss to follow-up.	95.6% (CI_95_, 94.5–96.7)	91.5% (CI_95_, 89.6–93.4)
HIV-RNA >50 copies/mL x2 or >50 x1 followed by loss to follow-up.	94.4% (CI_95_, 93.1–97.7)	85.2% (CI_95_, 82.9–87.5)
HIV-RNA >50 copies/mL x2 or >50 x1 followed by loss to follow-up + treatment change due to a single detectable viremia.	93.5% (CI_95_, 92.0–95.0)	83.5% (CI_95_, 81.0–86.0)

Among the 74 patients experiencing VF, the median plasma HIV-RNA was 752 copies/mL (range, 205–50,775). In 49 (66.2%) of those patients, the HIV protease could be amplified and results are shown in Table 4.

**Table 4:** New mutations in the HIV protease gene after VF on ritonavir-boosted protease inhibitors monotherapy (mtPI/rtv).

HIV protease gene after VF on mtPI/rtv (n = 49/74)	n (%)
Wild type	31 (63.3)
Only mm (M, 1; range, 1–3)	14 (28.5)
▪ 1mm (L10V, 1; I62V, 2; L63P, 3; I64V, 1; A71T, 1)	8 (16.3)
▪ 2 mm (L63P plus I62V or I64V or A71V or V77I or V82I)	5 (10.2)
▪ 3 mm (L63P, M46I, I62V)	1 (2.0)
1 MM + mm	3 (6.1)
▪ V32I	1 (2.0)
▪ I50V, L10I, K20R, M36I, M46I, G48V	1 (2.0)
▪ V82A, L10V, M36I, M46I, M54V	1 (2.0)
2 MM + mm	
▪ V32I, T74P, L76V	1 (2.0)

M: median; MM: major protease resistance mutations; mm: minor protease resistance mutations.

### HIV-RNA evolution during the monotherapy and the outcome after virological failure

**Fig 2:**
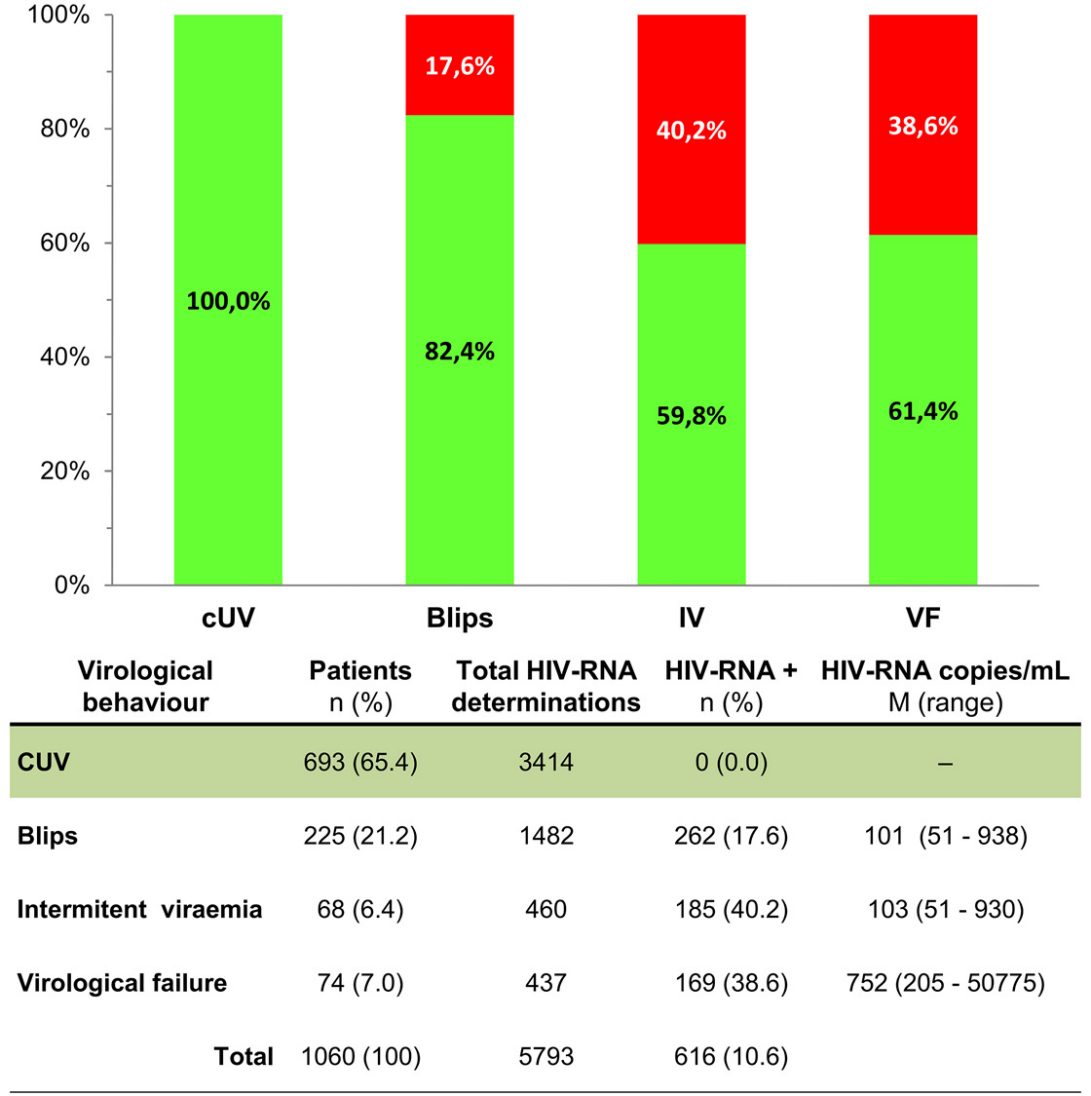
Percentages of detectable HIV-RNA determinations (red) according to virological behavior during the follow-up for ritonavir-boosted protease inhibitor monotherapy. Undetectable viremia (green).

The median HIV-RNA determinations per subject was 5 (IQR, 4–7; range, 1–12), which adds up to a total of 5,793 HIV-RNA determinations. In addition to the 74 patients who experienced VF (7%), 693 patients (65.4%) maintained viral suppression during the follow-up, 225 (21.2%) experienced blip episodes (1, 190 patients; 2, 33 patients; and 3, 2 patients), and 68 (6.4%) showed intermittent viremia with a median of 40.0% of detectable HIV-RNA determinations (range, 14.0–100%) (Fig 2).

One to three months after VF, only two out of 74 patients who presented a VF maintained a VL >50 copies/mL (1170 and 139 copies/mL, respectively). Sixty-four patients (86.4%) regained virological control either by continuing on mtPI/rtv and improving adherence (n = 22; 29.7%) or by adding one or two NRTIs (n = 36; 48.6%) or maraviroc (n = 6; 8.1%) to the protease inhibitor. Additionally, eight patients were lost to follow-up (10.8%).

## Discussion

The ART simplification to mtPI/rtv has raised great concern from the beginning. Indeed, these regimens have largely been called into question much more than triple therapy. Whereas the efficacy of the latter is assessed in terms of achieving good control of the viremia, in the case of mtPI/rtv its reliability to either control HIV reservoirs or penetrate "sanctuaries", such as the genitourinary tract or the CNS, has been extensively questioned, as has the potential higher incidence of neurocognitive impairment. However, currently most of these events have been favorably clarified [26–34].

The results in this large cohort are similar to or slightly inferior to those observed in the main clinical trials on mtPI/rtv [5–10], as they reflect the results obtained in usual clinical practice more than the results from selected patients inherent to clinical trials. Moreover, the majority of the patients in this cohort were not checked for tolerance to LPV/rtv or DRV/rtv before starting monotherapy and some of the patients with VF in our cohort might actually be dropouts, as suggested by the high viral load they presented at failure.

Until now, mtPI/rtv was only allowed for patients with no previous VF while on protease inhibitor-based regimens. We believed that the absence of major protease resistance mutations to LPV/rtv or DRV/rtv, respectively, would not affect the virological efficacy of mtPI/rtv in cases of an earlier VF on PI-based regimens. Indeed, our results show that neither a history of VF on PI-based regimens nor the presence of protease mayor resistance mutations not reducing susceptibility to the PIs used or minor resistance mutations, including those specific for LPV/rtv or DRV/rtv, negatively affected the efficacy of mtPI/rtv as a maintenance treatment. In fact, the latest European guidelines admits the possibility of using it whenever there are no resistance to the PI used [14].

Moreover, mtPI/rtv allowed for the simplification even of complex therapy due to extensive resistance, with a VF rate of only 1% in these patients. Other results of our series are also worth highlighting, such as the low incidence and grade of the AEs causing a treatment change, the favorable liver safety profile in a population in which more than 40% of patients are affected by chronic hepatitis C or cirrhosis, and the very low rate of new major and/or minor resistance mutations at VF, which barely compromised the activity of LPV/rtv or DRV/rtv, making it possible to regain virological control switching back to dual or triple therapy or even only improving adherence. Although some studies have shown that a CD4^+^ T-cell count nadir below 100–200 cells/μL and hepatitis C virus co-infection are factors associated with FV [9,35], but our findings do not support these observations nor a relationship with the previous time on virological suppression.

Nevertheless, our study has several limitations due to its retrospective nature and the lack of both a triple therapy comparator arm and a proper adherence assessment, particularly when adherence has been extensively associated with VF on mtPI/rtv (6,34,35). In contrast, the fact that all of the adult HIV-infected patients in our cohort switched from a triple antiretroviral regimen to mtPI/rtv at the participant centers minimized the selection biases, thereby reflecting what this simplification regimen can potentially offer in a real-world, clinical practice.

In summary, switching to mtPI/rtv is a feasible simplification option for virologically suppressed patients, even those with previous VF while on protease inhibitor-based regimens and with complex antiretroviral regimens, as long as no major resistance mutations are present for the administered drugs. A large proportion of patients maintained sustained viral suppression, and there was minimal risk of resistance development in those who failed.
